# Powdery Mildew Decreases the Radial Growth of Oak Trees with Cumulative and Delayed Effects over Years

**DOI:** 10.1371/journal.pone.0155344

**Published:** 2016-05-13

**Authors:** Didier Bert, Jean-Baptiste Lasnier, Xavier Capdevielle, Aline Dugravot, Marie-Laure Desprez-Loustau

**Affiliations:** 1 BIOGECO, INRA, Univ. Bordeaux, 33610 Cestas, France; 2 DRIAAF ILE DE France, F-94234 Cachan, France; 3 Inserm U1018—F-94800 Villejuif, France; Agriculture and Agri-Food Canada, CANADA

## Abstract

*Quercus robur* and *Q*. *petraea* are major European forest tree species. They have been affected by powdery mildew caused by *Erysiphe alphitoides* for more than a century. This fungus is a biotrophic foliar pathogen that diverts photosynthetate from the plant for its own nutrition. We used a dendrochronological approach to investigate the effects of different levels of infection severity on the radial growth of young oak trees. Oak infection was monitored at individual tree level, at two sites in southwestern France, over a five-year period (2001–2005). Mean infection severity was almost 75% (infected leaf area) at the end of the 2001 growing season, at both sites, but only about 40% in 2002, and 8%, 5% and 2% in 2003, 2004 and 2005, respectively. Infection levels varied considerably between trees and were positively related between 2001 and 2002. Increment cores were taken from each tree to assess annual ring widths and increases in basal area. Annual radial growth was standardised to take the effect of tree size into account. Annual standardised radial growth was significantly and negatively correlated with infection severity in the same year, for both 2001 and 2002, and at both sites. The decrease in growth reached 70–90% for highly infected trees. The earlywood width was poorly correlated with infection severity, but the proportion of latewood in tree rings was lower in highly infected trees (60%) than in less heavily infected trees (85%). Infection in 2001 and 2002 was found to have a cumulative effect on radial growth in these years, together with a delayed effect detectable in 2003. Thus, even non-lethal pathogens like powdery mildew can have a significant impact on tree functioning. This impact should be taken into account in growth and yield models, to improve predictions of forest net primary production.

## Introduction

The impact of abiotic factors on forest productivity constitutes a highly active broad field of investigation, particularly in the context of climate change [1, 2]. Dendrochronology analyses of tree-ring width provide an integrative approach to the assessment of this impact [3–7].

Far fewer studies have investigated the effects of biotic factors. Strong impacts of biotic factors have been reported for seedling or tree survival (e.g. [8–10]), and for tree growth in severe outbreaks of pest and pathogen infestations [11, 12]. More limited or chronic effects of biotic factors on tree growth have been investigated in only a few pathosystems, such as *Dothistroma* needle blight on *Pinus radiata* in New Zealand [13–14], Swiss needle–cast caused by *Phaeocryptopus gaeumannii* on *Pseudotsuga menziesii* in New Zealand [15] or North West America [16], *Armillaria* root rot on various conifers [14, 17, 18] or *Mycosphaerella* leaf blights on *Eucalyptus globulus* [19]. As regards *Quercus robur*, only the effects of defoliation by insects have been investigated on mature trees [20].

Relating pathogen infection to tree growth is especially important when dealing with forest plantations where disease translates into yield loss [13–15, 19]. However, decreased growth caused by pathogens can more generally affect all ecological functions and services provided by forests, in particular their role in biogeochemical cycles. Up to now, long-term growth reductions due to pathogens are not included in ecosystem models which could lead to inaccurate estimations of carbon uptake and storage [21]. Several authors have emphasized the need of “adding pests into the equation” for forest tree growth modelling [21–23]. Among various types of pathogen damage, foliar infections are likely the easiest to model a quantitative impact on tree growth since they directly reduce the photosynthetically active area of trees [21]. However, predicting the outcome of foliar attack on host growth is not so straightforward, because non-linear relationships between foliar infection and productivity losses are expected to occur at different scales [22–24]. Studies on whole leaves have shown that the decrease in photosynthesis resulting from leaf damage by parasites is generally greater than could be accounted for by the direct effects of the loss of leaf area alone [25–27]. On the other hand, compensatory effects may occur at the whole-plant level [23, 26]. More investigations are thus needed to improve our understanding of the relationships linking foliage damage and biomass production, and to enable pathogen damage to be included in forest tree growth modelling [21–23].

This study focuses on oak powdery mildew. Oaks, including the pedunculate oak *Quercus robur* L. and the sessile oak *Q*. *petraea* (Matt.) Liebl., are major tree species in European forests. They are widely distributed and are considered to be of high economic, cultural and environmental value [28]. Since the beginning of the 20^th^ century, European oaks have been affected by powdery mildew caused by *Erysiphe alphitoides* (previously known as *Microsphaera alphitoides*) and related species [29]. Powdery mildew fungi are obligate biotrophs that absorb nutrients from the living cells of their host plant, without killing them, through differentiated structures called haustoria [30]. They thus divert photosynthetate from the plant for their own nutrition [31]. Studies on the physiological effects of powdery mildew infection on oak leaves have reported negative effects of the disease on net CO_2_ assimilation rates [32], although the effects were moderate, with photosynthesis maintained even when the leaf surface was almost completely covered with mycelium [33]. Powdery mildew has particularly damaging effects on young seedlings, in nurseries, plantations and natural regeneration conditions, and on mature trees, in conjunction with other factors leading to decline [34]. Decreases and changes in growth have been reported in young pedunculate oak seedlings [35]. However, no estimates of growth loss in older moderately infected trees are currently available. The aim of this study was to assess the impact of powdery mildew on the radial growth of oak trees, by studying the relationship between levels of foliar infection and growth increments, using a dendrochronological approach.

## Materials and Methods

### Study Site and Oak Stands

Data was obtained from two *Quercus robur* L. field experiments in the Landes de Gascogne region in south-western France. The experiments were in community-owned forests and an agreement allowing samples to be taken from was signed with INRA. One experiment was located near Biscarosse (“B1 site” at 44°26N-1°04W, 40m a.s.l, S1 Fig) and the other near Pontenx-les-Forges (“P8 site” at 44°17N-1°04W). Oaks were planted in November 1994, after two years in a nursery. Each experiment site consisted of six rows of 17 oaks with 2 m between adjacent trees in each row and 3 m separating the rows. The rows corresponded to three geographic provenances of *Q*. *robur*: two rows were planted with seedlings raised from acorns collected in the Vouzeron forest (47°15N-2°13E) located 400 km northeast of the study site, two rows were of local provenance, from the Hermitage forest (44°44N-0°46W), and two rows were of local provenance originating from a forest 75 km further south, at Bias (44°08N-1°13W). Provenances were planted in unreplicated blocks at each site meaning that provenance effects and main-plot effects were confounded. However, the small size of each plot and the homogeneity of the field could be expected to reduce this disadvantage. This region has a temperate-maritime climate, with an annual mean temperature of 13.6°C and about 940 mm precipitation per year (mean: 1980–2010). P8 is drier than B1, because the hydromorphic sandy spodosol at this site has a relatively cemented spodic horizon (dense podzol) over 100% of the area at a depth of 43 cm, whereas the iron pan covers only 44% of the surface at B1 and is located at a depth of 58 cm.

### Monitoring of Infection and Tree Growth

Oak growth and powdery mildew infection were monitored at tree level at both sites in 2001 and 2002, using additional observations in 2003–2005 for the P8 site. Shoot growth phenology was monitored by weekly assessments in spring, with a seven-point scale: 0 = dormant bud, 1 = swollen bud, 2 = leaf tips extended beyond bud scales, 3 = first leaves unfolded, 4 = leaves unfolded but not yet full-size, 5 = leaves fully expanded but still tender and light green, 6 = mature leaves (dark green and tough). In 2001 at the B1 site and in 2002 at both sites, late frost caused leaf damage (mid-May assessment). The percentage of leaves affected was recorded on each tree.

Powdery mildew infection was assessed at tree level during the growing season, by trained observers. Initial (primary) infections occur in early spring as small whitish circular lesions on a few leaves, mostly visible on the upper leaf surface. Progressively, an increasing number of leaves are infected, and, as secondary infections occur, the whole leaf surface may be covered by mycelium and spores, resulting in a whitish appearance. The percentage of the leaf area affected by oak powdery mildew (infection severity) was estimated as follows. The leaves of each tree were examined by eye and assigned to one of four damage groups: “0” for leaves with no powdery mildew, “A” if less than 50% of the surface area of the leaves was infected, “B” if the infected area covered more than 50%, “C” for severely distorted and/or necrotic or dead leaves. The whole tree infection index (Inf) was then calculated as follows:

Inf=0.25(%leavesingroupA)+0.75(%leavesingroupB)+1(%leaves in group C)

Inf therefore varies from 0 to 1, corresponding to a percentage of infected leaf area from 0 to 100%.

In 2001, both sites were monitored nine times, from May 18^th^ to August 23^rd^. A mean infection index was calculated for the first flush, from the first four assessments until mid-June (“spring infection”). We also calculated another mean infection index for the end of June to the end of September (“summer infection”). In the other years, infection levels in the spring were much lower (see below) so assessments were carried out only during the summer.

### Dendrochronology

In April 2013, an increment core was obtained 0.30 m above ground level, from all the surviving oaks, on the main stem and up to two additional stems; individuals with a bushy appearance, with no main stem and more than three stems were discarded. The cores were planed with a cutter and digitalised on a scanner at a resolution of 1600 dpi. Ring widths were measured to the nearest 0.01 mm on the image, with dendrochronology software (Windendro^TM^, Regent Instruments Inc., Canada). The series were cross-dated with the Gleichläufigkeit index in Windendro software (i.e., the percentage of cases of agreement in the signs of the first-difference of two time series). We rejected the few samples with inner tree rings dating from 1998 or later due to apex death followed by re-sprouting, or for which the core missed the pith. This selection procedure resulted in the use of trees with at least three measured tree rings dating from before 2001. The final dataset consisted of cores from 67 oaks at the B1 site and 61 oaks at the P8 site.

The ring widths (in mm) were then converted into basal area increment (BAI in cm^2^) values, to provide a better estimate of total radial growth. For each year, the basal area (BA) was calculated as

$$BA_{n}=\pi \;r_{n}{}^{2}$$

with r_n_ the sum of ring widths from the pith to year *n* inclusive. Basal area increments (BAI) were then calculated as:

$$BAI_{n}=BA_{n}-BA_{n-1}$$

For multi-stem trees, the sum of BA_n_ for the different stems was calculated per year, and BAI_n_ was then calculated as if the tree had one stem. BA values ranged from 9 to 190 cm^2^ in 2012. The largest oak sampled therefore had a BA 21 times larger than that of the smallest oak.

### Data Analysis

The summer powdery mildew infection index was analysed using a linear mixed model including site, provenance, year (2001 and 2002) and their interactions as fixed effects and individual trees as a random factor. A preliminary analysis showed that there was no significant interaction between year and site or year and provenance. The infection model was therefore defined as follows:

$$Inf_{ijkt}=\;\mu +\alpha Site_{i}+\beta Provenance_{j}+\gamma Site*Provenance_{ij}+\delta Year_{t}+Tree_{ijk}+\varepsilon_{ijkt}$$ (1)

where Inf_ijkt_ is the summer mean infection index for site “i”, provenance “j”, tree “k”, in year “t,” μ is the overall mean. α, β, γ, δ are the parameters corresponding to the fixed effects of site, provenance, their interaction, and year, Tree_ijk_ is the random tree effect and ε_ijkt_ is the residual error such as Tree_ijk_ ~N (0, σ^2^_Tree_) and ε_ijkt_ ~N (0, σ^2^_ε_), and Tree_ijk_ are independent of ε_ijkt_ ∀ i,j,k,t and ε_ijkt_ are independent ∀ i,j,k,t.

Multiple comparisons of means were carried out between provenances at a single site, for a given year, with the Bonferroni test. Linear models were used to investigate the relationship between growth and powdery mildew infection. Initial tree size was included in the model because large trees generally grow faster than small ones.

We first analysed effects of infection on radial increments on an annual basis, for 2001 and 2002. Infection levels were too low in subsequent years to perform such analyses. The BAI_n_/BA_n-1_ ratio (expressed as a percentage) describing the standardised radial growth of the tree was used as the independent variable. It was assumed that there was a general linear relationship with no intercept between the basal area increment of one year and the basal area of the stem in the previous year (S2 Fig, [36]). This ratio takes into account the wide range of sizes of the trees studied and makes it possible to compare their growth during a given year (i.e., 2001 or 2002). We assumed that infection had a linear effect on this ratio, as described by the following model:

$$\frac{BAI_{n}}{BA_{n-1}}=\beta_{0}+\beta_{1}\;Inf_{n}+\varepsilon$$ (2)

where β_0_ is the intercept and β_1_ is the slope. As the value of the slope of the regression line depends on the range of BAI_n_/BA_n-1_, comparisons between years and between sites required the use of a standardised slope coefficient (SSC). When Inf increases by 1 unit, BAI_n_/BA_n-1_ decreases by β_1_ (actually its absolute value, assuming β_1_ is negative) and this decrease can be expressed as a percentage of β_0_:

$$SSC=100*\frac{\left|\beta_{1}\right|}{\beta_{0}}$$ (3)

For a given year, when SSC is 0.75, for example, oaks with an infection severity of 100% display 75% less growth than uninfected oaks. We used the delta method to computed 95% CI of SSC. The delta method approximates the standard errors using a first-order Taylor approximation [37].

We also analysed the cumulative effects of infection in 2001 and 2002 on growth over these two years, as follows:

$$\frac{BAI_{2001}+BAI_{2002}}{BA_{2000}}=\beta_{0}+\beta_{1}Inf_{2001+2002}+\varepsilon$$ (4)

with Inf_2001+2002_ the mean of the summer infection indices for 2001 and 2002

We further analysed the cumulative effects of infection by partitioning the whole sample of trees for the 2002 analyses into two subsamples with the median value of infection severity in 2001 used as the cut-off point. Model (2) for 2002 was then tested for the two subsamples.

Finally, we explored delayed effects of infection, by looking at effects on growth in the years following a season with infection. Our study period consisted of two successive years (2001 and 2002) with high powdery mildew infection followed by several years with low levels of infection (see below). We therefore assessed the cumulative effects of infection in 2001 and 2002 on radial growth in 2003 and subsequent years, as follows:

$$\frac{BAI_{n}}{BA_{2000}}=\beta_{0}+\beta_{1}Inf_{2001+2002}+\varepsilon$$ (5)

with *n* = 2003, 2004 and 2005.

For model (2), we determined whether the mid-May frost had an effect on the relationship between powdery mildew infection and radial growth, by running the model in 2001 for the B1 site for two equally distributed classes with and without frost damage.

Statistical analyses were carried out and graphs were produced using the lm(), lme() and deltamethod() functions in the nlme and msm libraries of R [38].

## Results

### Infection Monitoring

**Fig 1:**
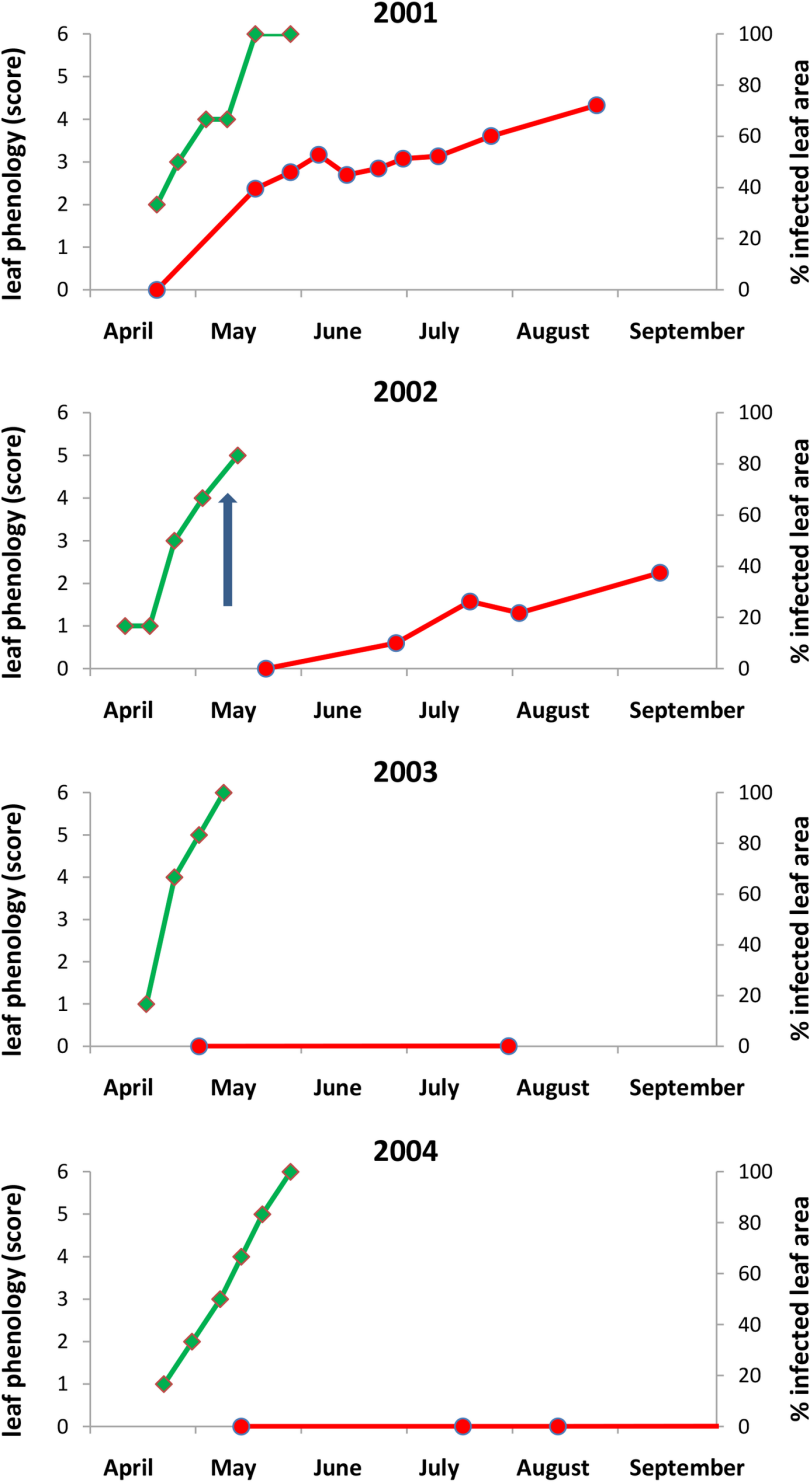
At the P8 site: the green line indicates phenology (median score, from 0 = dormant bud to 6 = fully developed leaves), and the red line indicates powdery mildew severity (mean % of the leaf surface infected). The blue arrow indicates the date of the late frost.

Budburst occurred during late April in all years (see Fig 1 for the P8 site). Very similar dynamics were observed at the two sites for all variables and years. Late frost damage was observed in mid-May in 2001 and 2002. In 2001, only the B1 site was affected, with 53% of trees and 30% of leaves affected on average. More generalised frost damage to leaves was observed in 2002, with 99% of trees affected at both sites, and a mean of 41% of the leaves frozen at B1, and 36% at P8.

**Fig 2:**
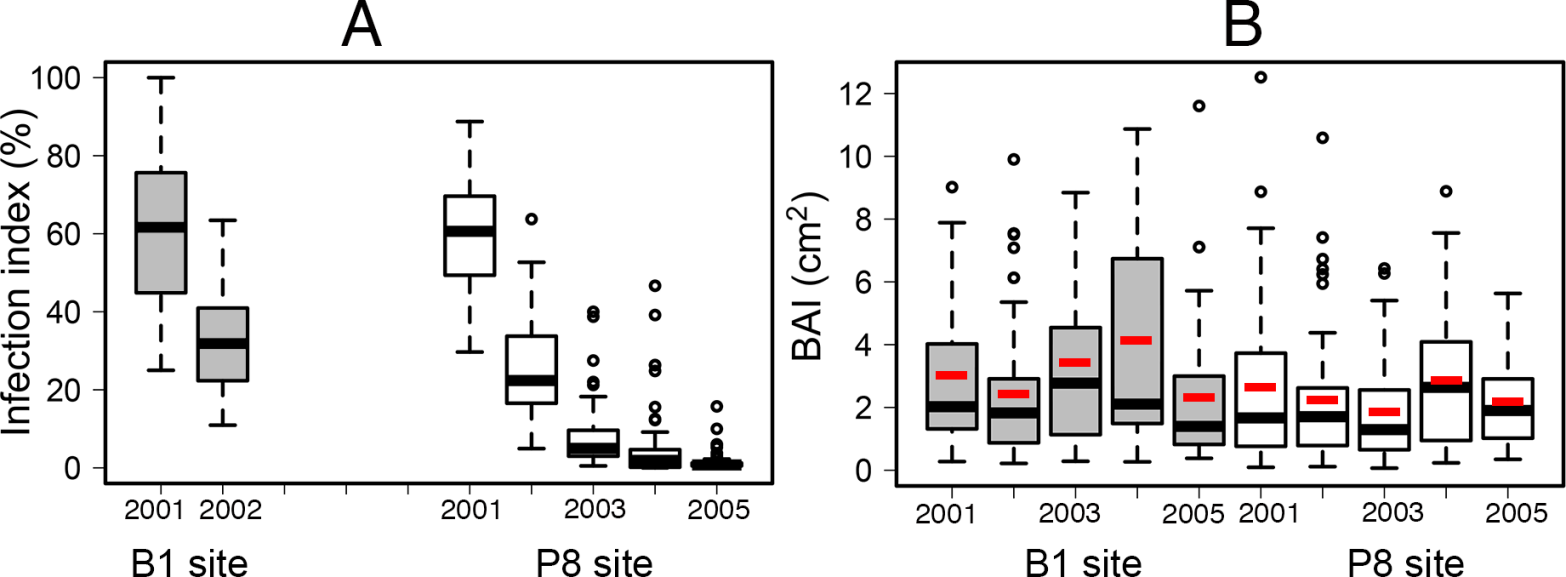
Final infection severity and basal area increment (BAI), by year and site. The boxplot shows the median in black, the mean in red, the first and third quartile, and the outliers. *n* = 67 oaks at B1 (grey) and *n* = 61 oaks at P8 (white).

In 2001, powdery mildew infections increased very rapidly after budburst and almost all leaves were infected at the end of May. Mean infection severity was estimated at about 50% of the leaf surface area during June-July and reached almost 75% at the end of the season, at both sites (Fig 1). Infection dynamics were slower, resulting in the persistence of infection at much lower levels, in subsequent years. In 2002, infection was delayed until refoliation after frost damage and progressed slowly through the summer, until a mean of about 40% of the leaf surface area was infected. No powdery mildew epidemics were observed in 2003, 2004 and 2005, resulting in very low final infection levels: 8%, 5%, and 2%, respectively (Fig 2A).

**Fig 3:**
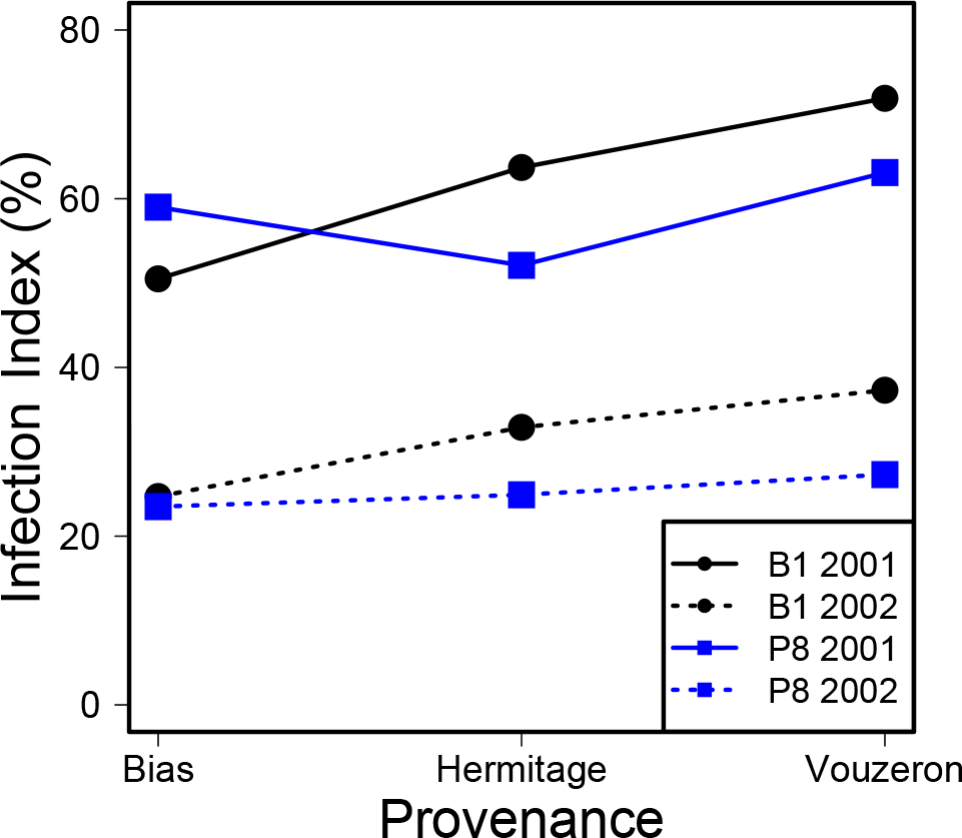
Mean infection index, by site, year and provenance. The black lines and dots are for the B1 site, the blue lines and squares are for the P8 site. The continuous and dotted lines are for 2001 and 2002, respectively.

The linear mixed model of infection (model 1) showed the following significant effects: site (*p* = 0.0157), provenance (*p* = 0.0002), year (*p*<0.0001), interaction between site and provenance (*p* = 0.0159), and tree effect (variance of the random tree effect, *p*< 0.0001). No significant difference in infection was observed between sites in 2001 with mean summer infection rates of 62% at B1 and 59% at P8 (*t* = 0.939, DF = 118, *p* = 0.349). However, a significant difference occurred in 2002, as infection rates were slightly lower at P8 than at B1 with a mean of 32% at B1 and 25% at P8 (*t* = 2.935, DF = 124, *p* = 0.004). The provenance effect resulted mostly from higher rates of infection among trees originating from the Vouzeron forest 400 km north of the study site than in trees with a local provenance, particularly those from Bias, with significant differences recorded at B1 (Fig 3; Table 1). The ranking of the Bias and Hermitage provenances differed between sites, resulting in a significant interaction between site and provenance in the analysis of variance. The strongest effect in the model was that of year, due to a large difference in mean infection levels between years (Figs 2A and 3).

**Table 1:** Number of trees, mean infection index and standard deviation, by site, provenance and year. The means in bold differed significantly (*p*<0.05) for the year concerned.

			B1 site			P8 site	
Year	Provenance	n	Mean	ɾ	n	Mean	σ
2001	Bias	22	**50.49**	16.24	24	59.02	15.39
2001	Hermitage	21	63.71	20.26	13	52.14	15.82
2001	Vouzeron	23	**71.87**	19.27	24	63.15	13.02
2002	Bias	22	**24.66**	9.22	24	23.49	9.72
2002	Hermitage	21	32.86	12.54	14	24.89	12.17
2002	Vouzeron	21	**37.35**	10.98	24	27.28	13.66

**Fig 4:**
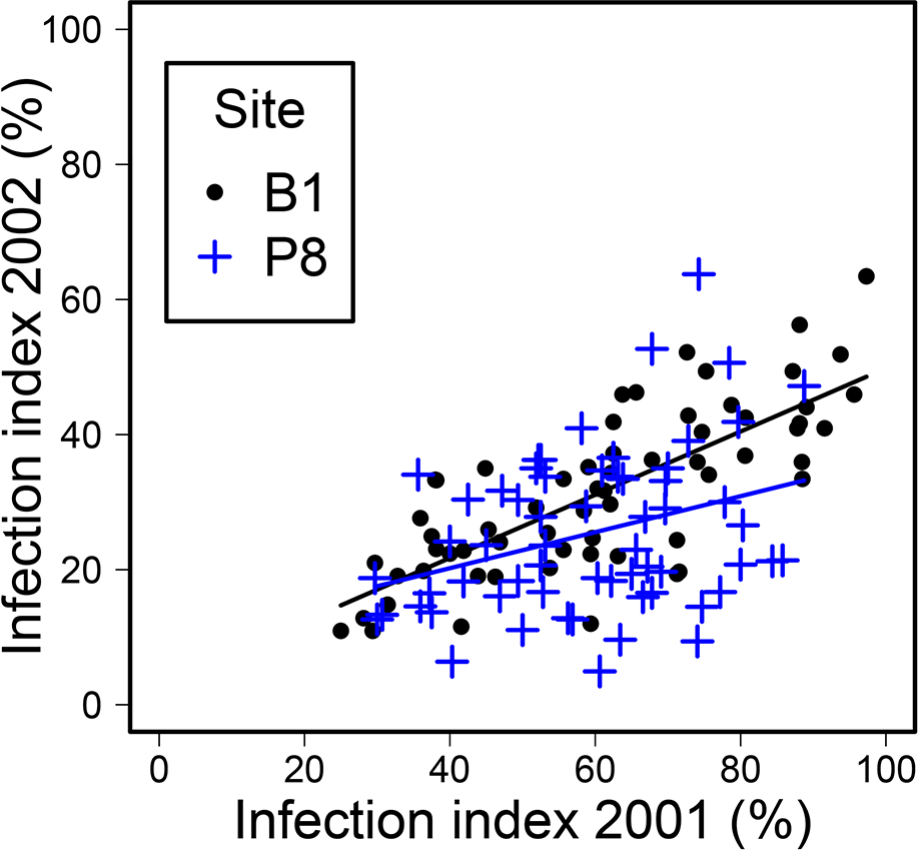
Infection severity in 2002, as a function of severity in 2001, at the two sites.

Tree infection levels were positively related between years (Fig 4). The Pearson coefficient was 0.764 (*p*<0.0001; df = 61) for the B1 site and 0.333 (*p* = 0.0087; df = 59) for the P8 site. At the P8 site, trees with lower infection levels in 2001 also showed low levels of infection in 2002, whereas highly infected trees in 2001 displayed a wide range of infection level in 2002.

### Effects of Infection on Growth

Soil characteristics differed between the sites, with a lower potential for growth at the P8 site. The oaks at P8 had a mean basal area only 50–70% of that at B1, from 2000 to 2005. The basal area increment (BAI) varied from 2 to 3 cm^2^ over years and sites, with large differences between trees. It was significantly lower at the P8 site than at the B1 site in 2000, 2003 and 2004 (Fig 2B).

**Fig 5:**
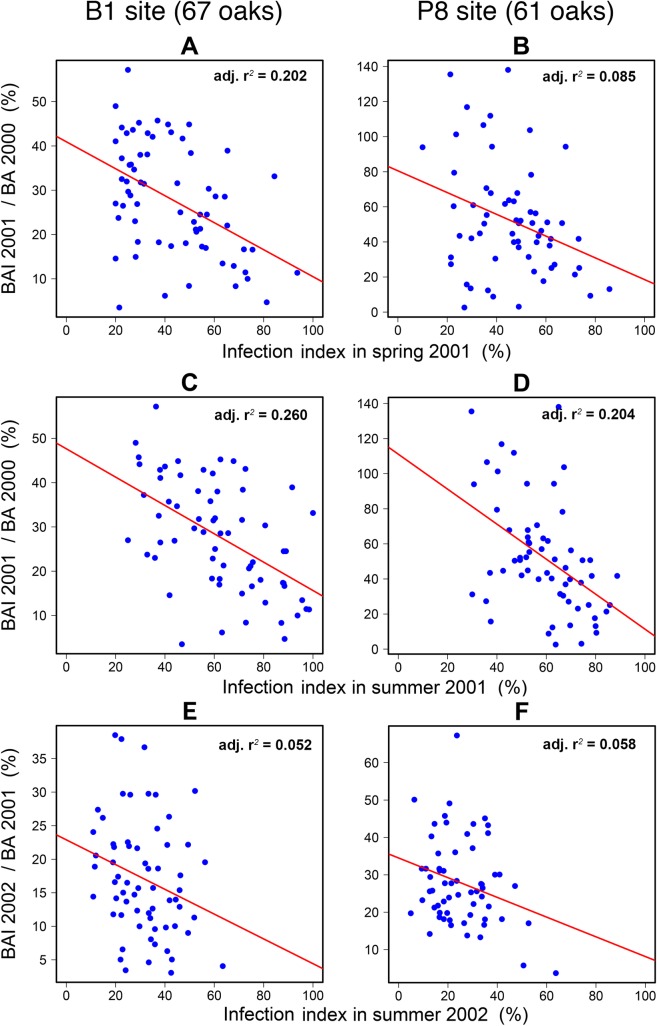
Growth index BAI_n_/Ba_n-1_ as a function of infection index for two years and two sites.

Standardised radial growth was significantly negatively correlated with infection severity in 2001 and 2002, at both sites (Fig 5). In 2001, the correlation with spring infection severity (Fig 5A and 5B) was weaker than that with summer infection severity (Fig 5C and 5D), at both sites (Table 2). For this year, the earlywood and latewood widths were distinguished within the annual ring, and the latewood/total ring width ratio was then calculated as a percentage. The correlation between this ratio and powdery mildew infection in 2001 was negative and highly significant: B1 site r = -0.509, *p*<0.0001 (S3 Fig) and P8 site r = -0.397, *p =* 0.0009. The observed growth reduction concerned principally the latewood, as earlywood width remained fairly constant. The correlation between earlywood width and summer infection was significant for the B1 site (r = -0.260, *p* = 0.0348) but not for the P8 site (r = -0.141, *p* = 0.259). Tree rings consisted of 85% latewood in trees with lower levels of infection but only 60% latewood in highly infected trees.

**Table 2:** Statistics for two sites and two years of powdery mildew infection. Regression coefficients (intercept and slope of regression lines in figures), slope probability (*p*), adjusted r², 95% confidence intervals, standardised slope coefficient (SSC, see Eq 3), 95% confidence interval of SSC assessed with Delta method [37], and Pearson correlation coefficient (r). Note: r and the slope have equal probabilities.

Fig	Dependent variable	Infection period	Site	Intercept	Slope	*p* slope	Adj r^2^	SSC	±95% CI	r
5A	BAI2001/BA2000	Spring 2001	B1	40.948	-0.305	<0.0001	0.202	0.745	0.242	-0.463
5B	BAI2001/BA2000	Spring 2001	P8	80.536	-0.620	0.0129	0.085	0.770	0.382	-0.317
5C	BAI2001/BA2000	Summer 2001	B1	47.645	-0.320	<0.0001	0.260	0.672	0.161	-0.521
5D	BAI2001/BA2000	Summer 2001	P8	111.133	-0.997	0.0001	0.204	0.897	0.212	-0.466
5E	BAI2002/BA2001	Summer 2002	B1	22.889	-0.185	0.0385	0.052	0.806	0.562	-0.259
5F	BAI2002/BA2001	Summer 2002	P8	34.511	-0.264	0.0343	0.058	0.764	0.560	-0.271
6A	(BAI2001+BAI2002)/BA2000	Mean 2001&2002	B1	87.268	-0.788	<0.0001	0.218	0.903	0.247	-0.480
6B	(BAI2001+BAI2002)/BA2000	Mean 2001&2002	P8	207.088	-2.622	<0.0001	0.277	1.266	0.246	-0.538
7	BAI2002/BA2001 Oaks with Inf 2001 ≤ 60.5	2002	P8	33.680	0.020	0.93 NS	0.035	0.060	1.333	0.017
7	BAI2002/BA2001 Oaks with Inf 2001 > 60.5	2002	P8	28.175	-0.230	0.0386	0.110	0,817	0.576	-0.373
8A	BAI2003/BA2000	Mean 2001&2002	B1	51.945	-0.436	0.0046	0.110	0.839	0.349	-0.352
8B	BAI2003/BA2000	Mean 2001&2002	P8	69.318	-0.730	0.0054	0.109	1.052	0.405	-0.352
NA	BAI2004/BA2000	Mean 2001&2002	B1	52.959	-0.296	0.145 NS	0.019	0.559	0.553	-0.186
NA	BAI2004/BA2000	Mean 2001&2002	P8	21.615	1.090	0.072 NS	0.038	5.043	17.181	0.232
NA	BAI2005/BA2000	Mean 2001&2002	B1	25.719	-0.093	0.376 NS	0.003	0.363	0.665	-0.113
NA	BAI2005/BA2000	Mean 2001&2002	P8	54.987	-0.127	0.629 NS	0.013	0.230	0.840	-0.063

The data were highly dispersed, but the relationship between infection and radial growth could be approximated by a linear regression over the range of infection severity, with no obvious threshold (Fig 5). The SSC was 0.7–0.9, with a mean of 0.785 in the case of severe summer infection in 2001 and 2002 (Table 2). The loss of radial growth may therefore be close to 78.5% at very high levels of powdery mildew infection, regardless of the site and the year studied.

**Fig 6:**
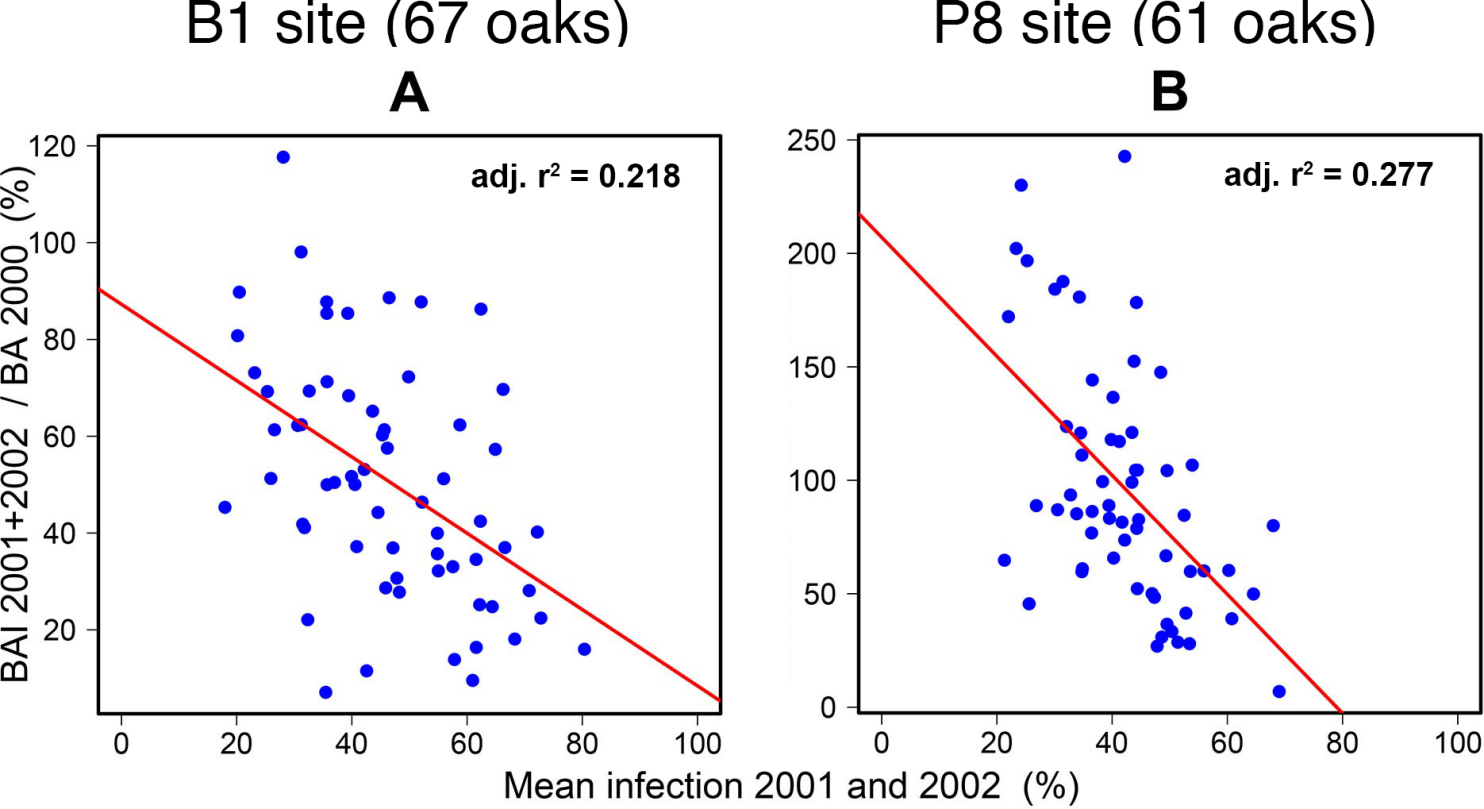
Growth index as a function of mean infection index in 2001–2002 and regression lines for two sites.

The cumulated effects of infections over 2001 and 2002 on cumulated radial growth for these two years were even stronger than the annual effects estimated separately. Very high standardised slope coefficients of 0.90±0.25 (95% C.I.) at Site B1 and 1.27±0.25 at Site P8 were obtained, corresponding to more than 90% growth reduction for 100% infection over the two years (Fig 6A and 6B; Table 2).

**Fig 7:**
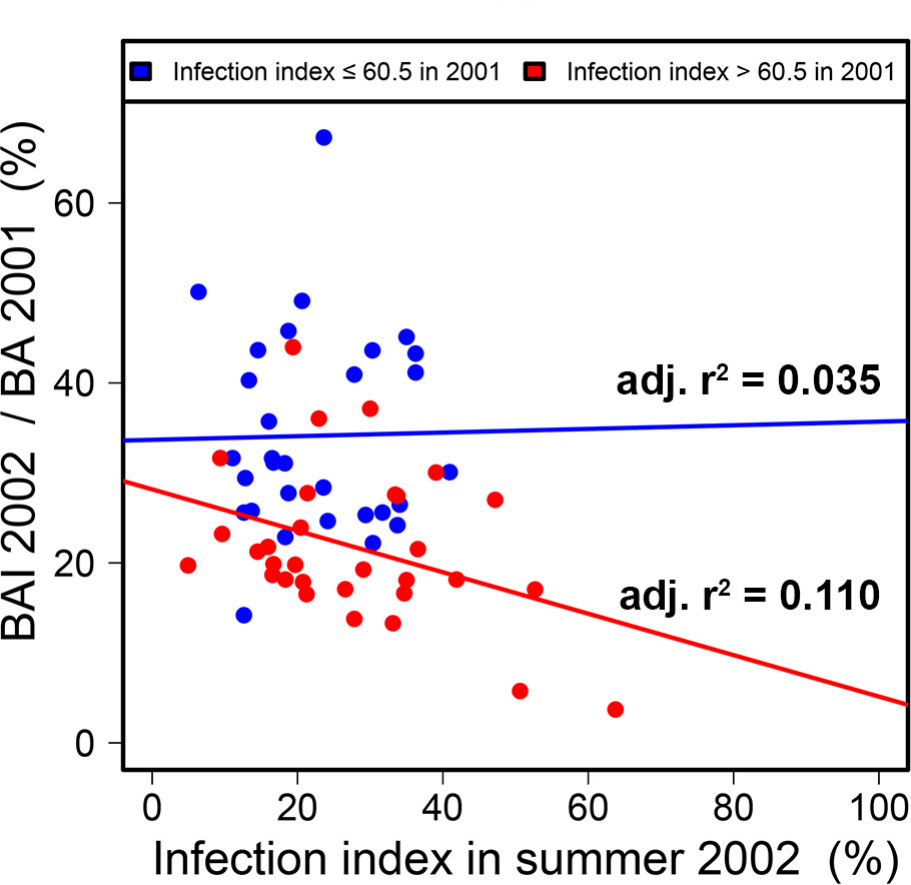
Growth index according to infection index in 2002, split into two groups on the basis of infection index for 2001 (low or high) at the P8 site.

The cumulative effect on radial growth of infection in 2001 and 2002 was also demonstrated by the observation that the effect of infection in 2002 on radial growth in the same year depended on the level of infection in the previous year at the P8 site (Fig 7; Table 2). Only trees highly infected in 2001 displayed a significant relationship between infection and radial growth in 2002. A similar analysis was not possible at the B1 site because the strong correlation between infection levels in 2001 and 2002 meant that trees highly infected in 2002 were invariably also highly infected in 2001.

**Fig 8:**
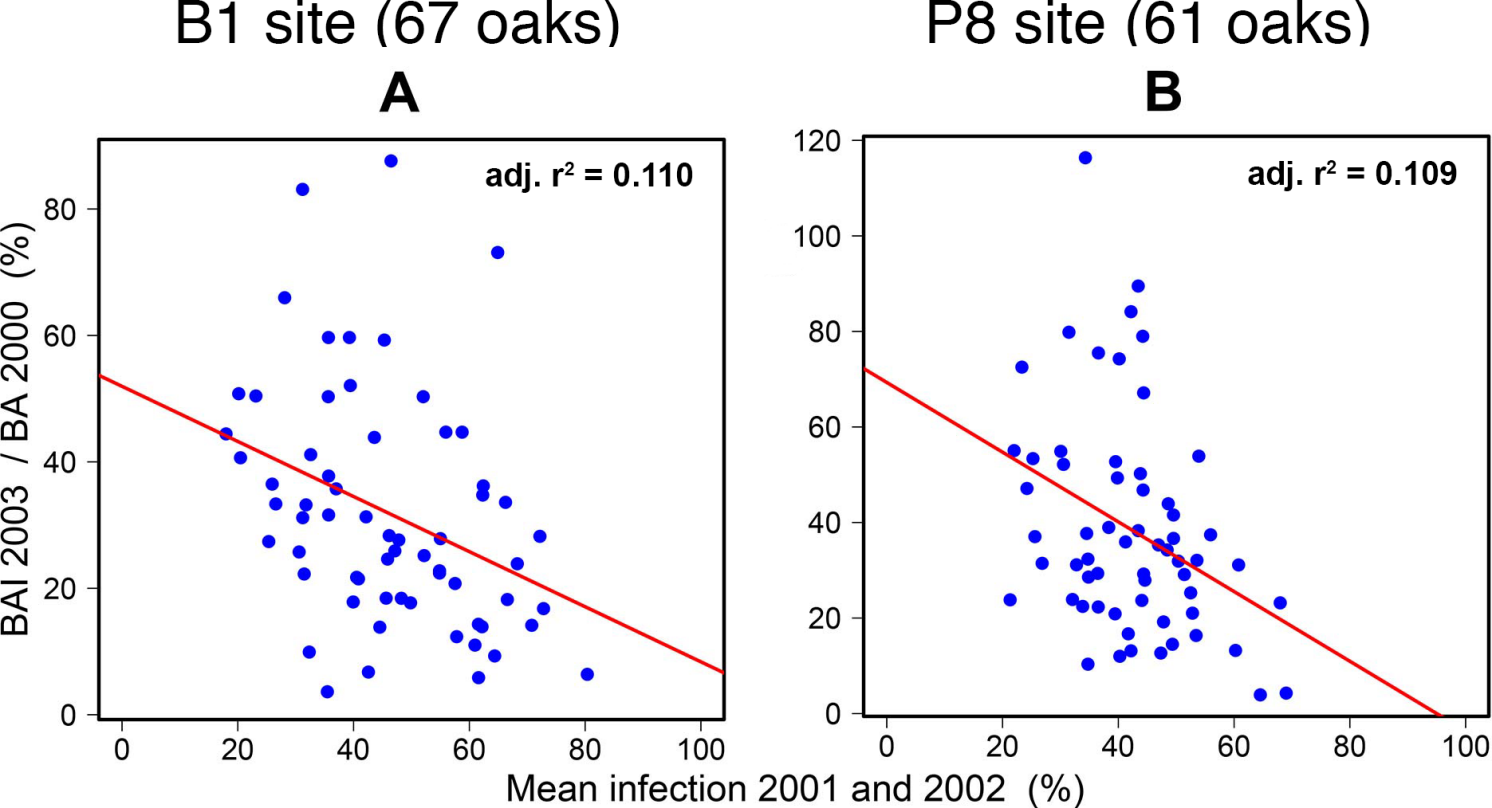
Growth index in 2003 as a function of mean infection index in 2001–2002 for two sites.

We also observed a cumulative and delayed effect of infection in 2001 and 2002 on the radial growth achieved in 2003 (Fig 8). No further effects on radial growth were observed in 2004 and 2005 (Table 2).

## Discussion

The impact of pathogens (and pests) on forests has been well documented for severe epidemics or outbreaks [9, 39, 40]. In addition to pathogen-induced mortality, impacts on tree growth have been assessed by comparing epidemic and non-epidemic years [41], or affected and healthy reference trees in the same diameter class [16] or infected trees and fungicide-treated controls [42, 43]. However, the more chronic impact on tree growth of moderate levels of infection has been investigated in less detail. In particular, functions describing the growth response to various intensities of infection at stand or tree level have been produced for only a few pathosystems, such as Swiss needle cast on Douglas fir in particular [14, 19, 44, 45].

We carried out detailed monitoring of powdery mildew infection in two young *Quercus robur* plantations in which natural epidemics occurred in two successive years, producing considerable variation in the level of infection between trees. Infection was assessed at individual tree level, by trained observers using a combination of disease prevalence (frequency) and severity (classes) criteria. *Erysiphe* species are epiphyllous. Infection is therefore conspicuous and visible symptoms are assumed to be strongly correlated with the amounts of tissue infected. We were therefore able to obtain a quantitative estimate of the percentage of the leaf area affected by the fungus for each tree. The validity of this assessment method was evaluated in preliminary trials (unpublished data). The almost Gaussian distribution of the tree disease severity values obtained for years with high levels of infection in this study and the significant positive correlations between trees obtained for independent assessments within and between years also suggest that this assessment method was appropriate. We used a dendrochronological approach to study the impact of infection on growth. This approach is useful for the reconstruction of growth dynamics over several years. Previous studies based on such an approach have compared the temporal growth dynamics of a particular species periodically affected by a pest or pathogen with those of a reference species growing in the same location but not susceptible to the pest or pathogen concerned [46, 47]. However, these studies focused mainly on temporal patterns of infection and growth. Other studies have addressed the effect of a pathogen on the radial growth of trees by taking into account the stem size distribution or growth history [48]. The healthy or infected trees were often paired together on the basis of stem size or competition level, resulting in discrete classes, particularly for root pathogens [49, 50]. We aimed to assess the effect of infection over a continuous range. We therefore adjusted for initial tree size in each year, making it possible to use the whole range of stem size for the sample studied without the need to pair individuals or to form classes.

This study of more than one hundred trees at two sites clearly demonstrated a significant negative effect of powdery mildew infection on the radial growth of oak trees. This negative effect was demonstrated in two years (2001 and 2002), differing in infection level and climate. In 2001, severe oak powdery mildew epidemics occurred throughout southwestern France [51], but the spring and summer precipitation was high which was beneficial to growth (Fig 2). Such conditions proved favourable for showing a significant effect of infection. In 2002, the growing season was rather dry, resulting in lower growth, and infection severity was lower, but between-tree variability remained high at our experimental sites and there was also a significant infection effect. A potential cumulative effect of leaf frost damage and powdery mildew infection might partly account for the observed growth patterns in our experiment, because late frost and powdery mildew occurred together in three of the four combinations of site and year situations studied, and the oaks with the highest levels of infection were also those displaying frost damage (S4 Fig). However, in 2001, the powdery mildew-growth relationship was similar between P8, where no frost damage was observed, and B1, where significant frost damage occurred. High levels of powdery mildew infection associated with oak refoliation after frost damage have frequently been reported [34]. In our experiment, frost damage may therefore exert its deleterious effects principally by increasing rates of powdery mildew infection, without modifying the effect of infection on tree growth.

The quantitative impact of *E*. *alphitoides* on oak growth has previously been described only for seedlings (reviewed in [34]). For example, height increment losses of 30–40% were observed in an experiment in south-west France in which naturally infected seedlings (about 30% of leaf area infected) were compared with control seedlings treated with fungicide [35]. Soutrenon [52] also reported large benefits of experimental fungicide treatments in naturally regenerating oak, in terms of seedling survival and biomass (greater increases in height and diameter than for untreated seedlings). The impact of foliar pathogens on the growth of older trees has been documented for other pathosystems. A 50–70% decrease in growth was reported following *Phaeocryptopus gaeumannii* infections in Douglas fir stands in southern Germany (Merkle 1951 in [14]), and a 27% decrease in basal area increment was reported in New Zealand [14]. Eucalypt growth following attacks by several foliar pests and pathogens has also been shown to be highly variable, with no effect until approximately 50% defoliation was reached in some cases, but a significant effect with as little as 10% defoliation in other situations [21].

In this study, a linear model fitted the data well for both years and sites, suggesting a proportional effect of infection (expressed as the percentage of the leaf area affected) on radial growth and the absence of a threshold effect. This contrasts with the findings of other studies, in which only high levels of infection were shown to affect growth [21, 53]. For example, the effects of the foliar pathogen *Dothistroma pini* on the growth of radiata pines were observed only at infection severities exceeding 20–30% in three- to four-year-old *Pinus radiata* trees [53, 54]. The growth reduction caused by two other needle cast diseases, *Lophodermium* sp. on pines and *Phaeocryptopus gaumannii* on Douglas fir, has also been shown to be less than 1:1 with respect to foliar disease indices [53]. However a more detailed analysis of our data also suggested that moderate levels of infection in a single year may have a limited effect. Linear models fitted the data particularly well (high R^2^) at both sites in 2001, when infection levels ranged from 30% to 100%. In 2002, with infection severity in the range 10–50%, an impact on growth was observed only on trees already severely affected in 2001 at the P8 site.

This cumulative effect of infection in successive years was clearly shown by even stronger infection-growth relationships in the analyses based on a two-year period than in separate estimations of annual effects. Very high standardised slope coefficients were obtained, exceeding 0.9 and corresponding to a 90% growth reduction for 100% infection over the two years. A similar effect of successive defoliations by oak herbivore insects on the relationship between increment loss and defoliation index has been reported [20]. The cumulative effect of infection in two successive years may be accounted for by delayed effects of the infection observed in 2001 on growth in the following year. First-order autocorrelations have been shown to be strong in tree-ring series in *Quercus*, because the climate in year_n_ also determines some of the growth in year_n+1_ [55]. Similarly, we observed carry-over effects of infection on growth in subsequent years. The strong correlations between infection rates in 2001 and 2002 made it difficult to separate the effects of these two years, although we were able to go some way towards this goal at P8, as discussed above. Nevertheless, use of the whole sequence of years, from 2001 to 2005, showed that infection in 2001–2002 had an effect until the end of the 2003 growing season. The absence of a relationship to tree growth in subsequent years suggests that trees recover after one or two years, as often reported for insect defoliations [12]. These findings also suggest that growth loss was a direct effect of powdery mildew infection and not an artefact due to inherent growth differences between trees displaying different levels of disease severity.

The cumulative effects of infection are probably favoured by the consistent behaviour of trees between years, as shown by the strong correlation between the infection levels in 2001 and 2002 for individual trees, and the significant tree effect in the infection model. Several factors may explain such individual effects. We observed a provenance effect, with higher levels of infection on trees originating from Vouzeron, the provenance furthest away from the experimental site. The greater resistance of local provenances may be accounted for by better local adaptation of oaks, although the pathogen has been shown to benefit more from local adaptation than the host in this pathosystem [56, 57]. Genetic effects on tree susceptibility to disease are likely, in the form of direct effects [35] or through host phenology, a highly heritable trait [58] that also influences vulnerability to late frost [59].

The effects of disease on growth depend on the mechanisms connecting infection to growth processes at various temporal and spatial scales, from the physiological processes in infected tissues to processes at tree level. For example, the impact of conifer foliar pathogens is highly dependent on the age classes of the needles infected, conditioning their contribution to photosynthesis [53]. The pattern of infection/ defoliation within a tree is also very important for determining growth responses and may contribute to the differences in such responses between trees of different ages [21, 41]. Infection also impairs growth by other more complex and diverse mechanisms, such as foliage retention, the occlusion of stomata, changes in light penetration, foliar nutrient translocation, gaseous exchanges, and carbohydrate dynamics [21, 26, 45, 53, 60–62]. The impact of broadleaf foliar biotrophic pathogens, such as powdery mildews, should be directly related to the percentage of the leaf area infected, particularly in young trees, because these pathogens develop on photosynthetically active leaves and hijack photosynthate for their own development via their haustoria, which tap into living leaf cells [30, 63, 64]. *E*. *alphitoides* has been shown to decrease the net CO_2_ assimilation rate in infected leaves [31, 33], through several mechanisms, including decreases in stomatal conductance [33] and the quantum efficiency of photosystem II [65]. Changes in carbohydrate translocation patterns, lower rates of photorespiration and higher rates of dark respiration have also been demonstrated [31–33]. The effect of foliar pathogens on the net photosynthetic rate of leaves usually exceeds that predicted from the lesion area, because the physiology of the remaining green leaf tissue is also affected [24]. Compensatory mechanisms in the healthy parts of the leaf may account for the impact of lesion size being smaller than expected in some cases ([25] for *Chrysomyxa rhododendri* on *Picea abies*). By contrast, changes in translocation due to heavy infection can lead to cell death and leaf shedding, resulting in an even greater carbon requirement for crown restoration [64].

Interannual variation in the radial growth of oaks in relation to climate has been shown to be due mostly to a decrease in latewood production during dry periods, with the production of earlywood remaining fairly constant [55]. Rubtsov [20] also reported a closer relationship of latewood increment than of earlywood increment to insect defoliation. Similarly, we show here that the effect of powdery mildew infection was principally due to a decrease in latewood width in the 2001 tree ring. Moreover, the impact on annual growth was more strongly related to summer infection (overall severity) than to spring infection (precocity of infection). This may be because the large vessels of the early wood are formed two weeks before budburst and are probably constructed from the carbon pool stored during the previous growing season [66]. The intensity of infection over the whole season therefore seems to be more important than the timing of infection, in terms of the impact on annual tree growth. The change in earlywood/latewood ratio in infected trees also indicates that powdery mildew has both qualitative and quantitative effects on wood production, as wood density depends on the proportion of latewood [67].

## Conclusion–Implications

Unlike root pathogens, which spread throughout a forest stand and persist indefinitely, foliar pathogens, such as *E*. *alphitoides* cause peaks of infection only in some years. Cyclic infestations have been reported for several pest insects [68]. In pathogens, between-year variations have often been shown to be related to climate variability, favouring fungus and disease development to different extents [69]. This is particularly true for oak powdery mildew, epidemics of which have been shown to be correlated with mild winters, such as that in 2001 [34]. In the context of global warming, favourable years for infection are therefore likely to become more frequent, increasing the impact of powdery mildew. Furthermore, this impact is likely to be increased by interactions with abiotic stresses, such as frost and drought, which are also likely to increase in frequency. The impact of pathogens should therefore be included in growth and yield models, to improve predictions of forest net primary production and carbon balance [22, 27, 39].

## Supporting Information

S1 Fig Map of France showing the two study sites (yellow and blue dots) and three oak provenances (red dots). Map plotted with R software.
(TIF)

S2 Fig For both sites, Basal Area Increment in 2001 according to Basal Area of the tree at the end of year 2000. The oaks were split in two groups of low or high infection when they were less or more infected than 63.8 or 62.9%, for B1 or P8 respectively. The regression lines give the relationship between the annual radial growth and the size of the tree. The model was: BAI = μ + αBA + β(BA * Infection) + ε. BA effect and the interaction between BA and Infection were significant (p<0.0001) for both sites, i.e., the slopes were different because the infection decreased significantly the radial growth all along the tree size range.
(TIF)

S3 Fig Percentage of latewood in the 2001 tree ring as a function of infection levels in 2001 for the B1 site. (TIF)

S4 Fig Growth index according to infection in 2001, for oaks with and without frost damage in spring 2001. The crosses indicate the means for the two groups.
(TIF)

S1 Table Data: one line per tree. Columns: Site, Provenance, Line number in the plot, Oak number in the line, Frost index in 2001 and 2002, Mean Infection index from May 18 to June 22 in 2001, Mean Infection from June 29 to August 23 in 2001 (taken as Infection index in 2001 in the data analyses), Infection index per year over 2002 to 2005, Basal Area per year over 1994 to 2012, Basal Area Increment per year over 1994 to 2012.
(XLSX)
