# Supplementary figures and images for: Powdery Mildew Decreases the Radial Growth of Oak Trees with Cumulative and Delayed Effects over Years
Source: PLoS One. 2016 May 13;11(5):e0155344. doi: 10.1371/journal.pone.0155344 (PMC4866782; doi:10.1371/journal.pone.0155344)

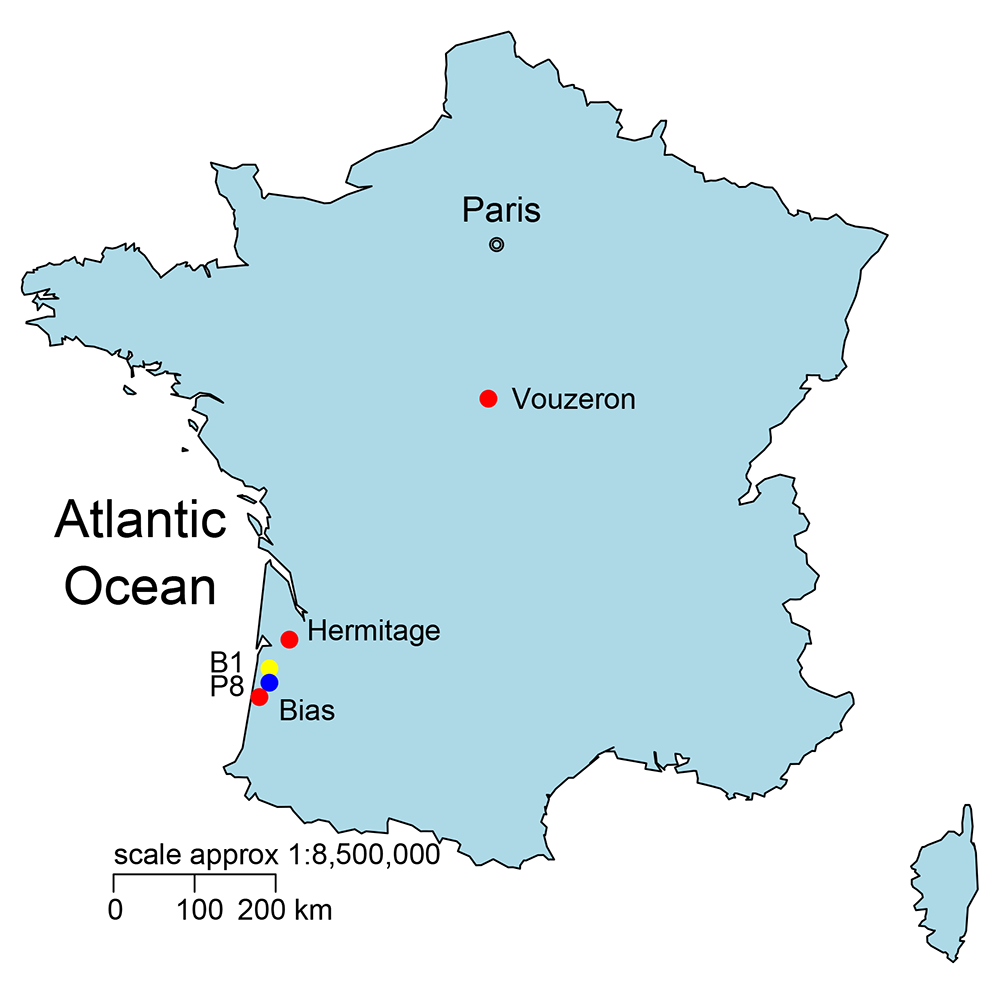

Supplement: S1 Fig — Map plotted with R software. (TIF) [file pone.0155344.s001.tif]

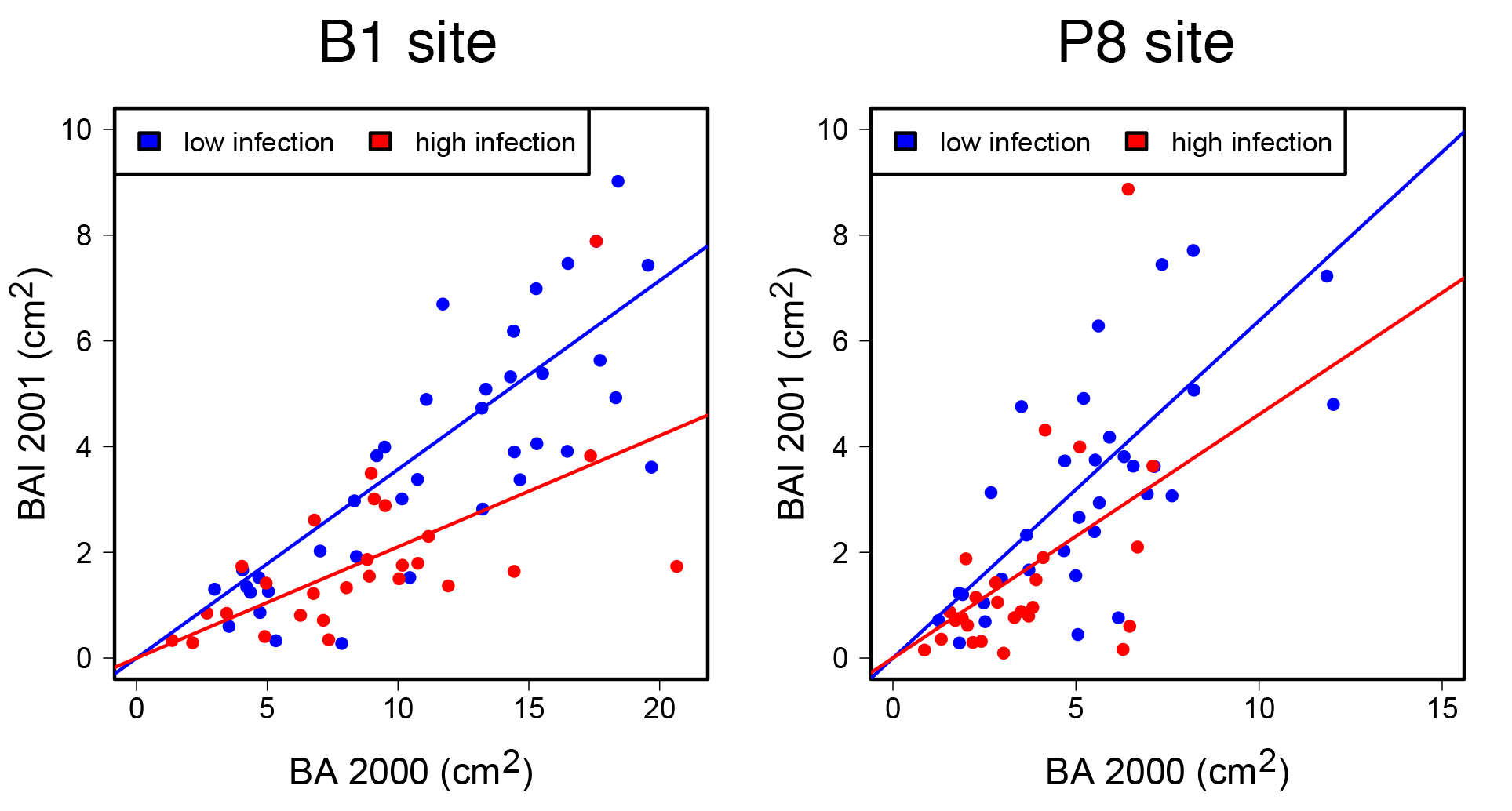

Supplement: S2 Fig — The oaks were split in two groups of low or high infection when they were less or more infected than 63.8 or 62.9%, for B1 or P8 respectively. The regression lines give the relationship between the annual radial growth and the size of the tree. The model was: BAI = μ + αBA + β(BA * Infection) + ε. BA effect and the interaction between BA and Infection were significant (p<0.0001) for both sites, i.e., the slopes were different because the infection decreased significantly the radial growth all along the tree size range. (TIF) [file pone.0155344.s002.tif]

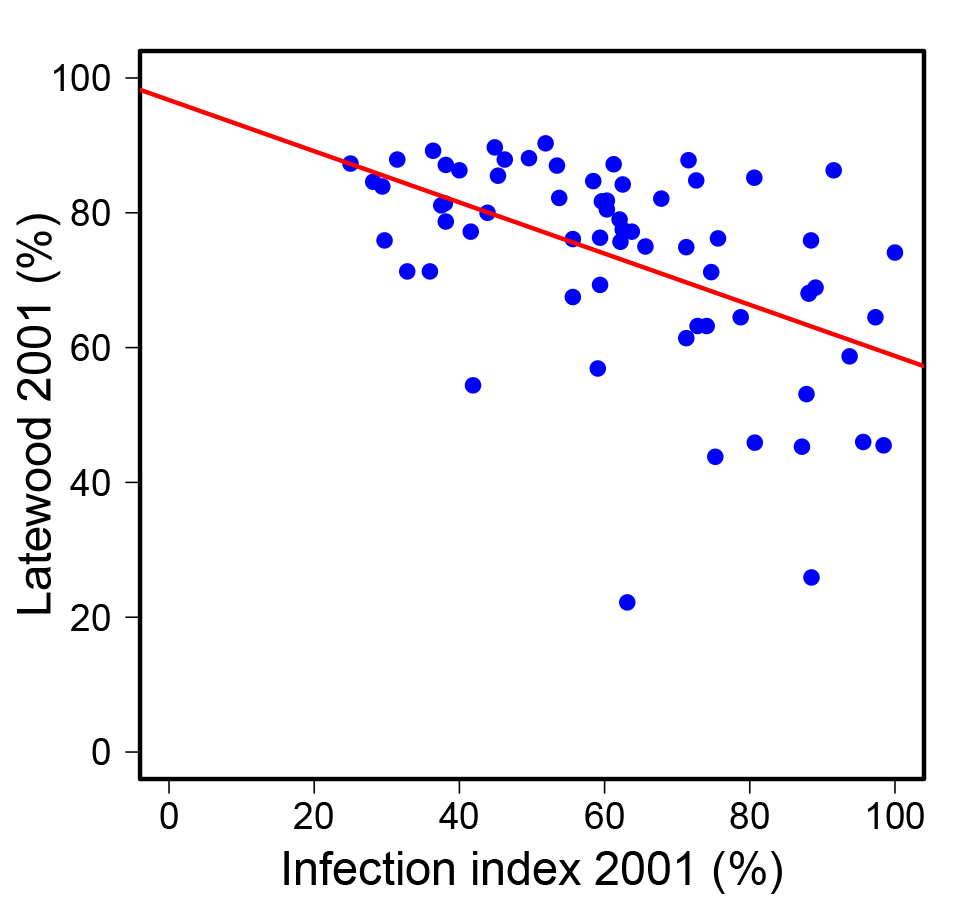

Supplement: S3 Fig — (TIF) [file pone.0155344.s003.tif]

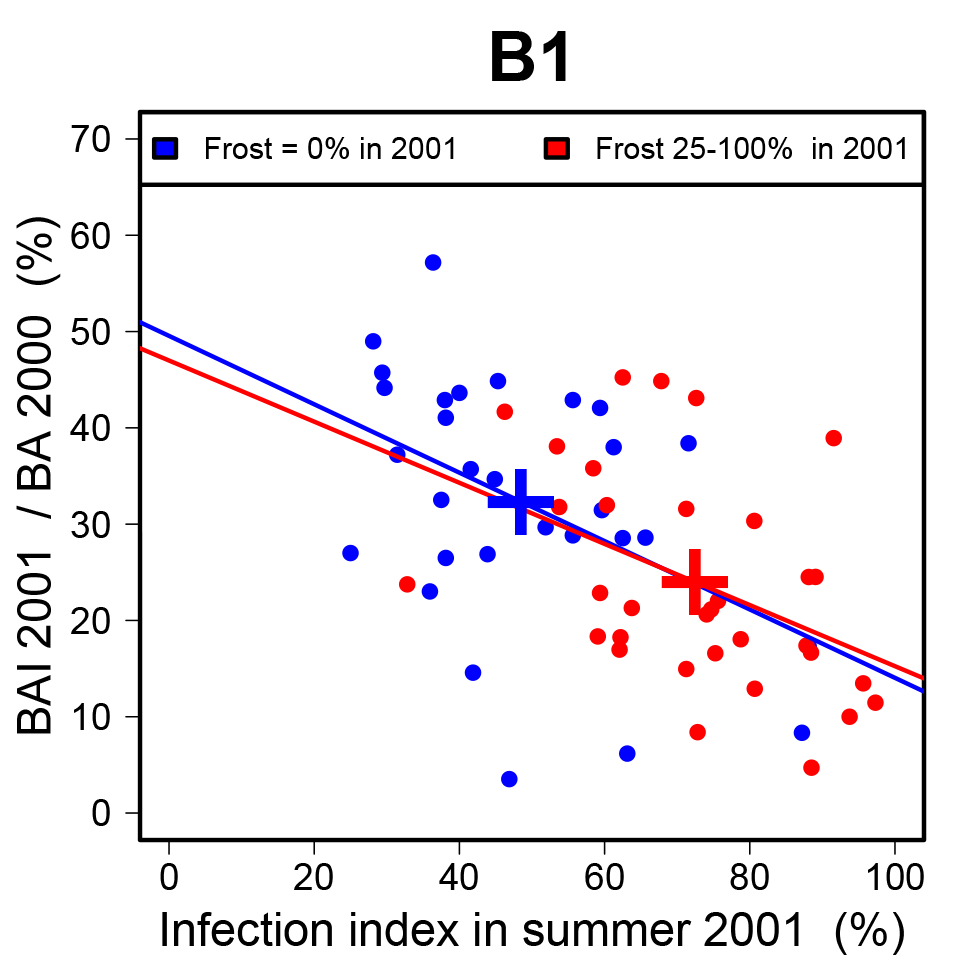

Supplement: S4 Fig — The crosses indicate the means for the two groups. (TIF) [file pone.0155344.s004.tif]
